# Ethyl 3,6-di-*O*-benzyl-2-de­oxy-*N*-phthalimido-1-thio-β-d-glucopyran­oside

**DOI:** 10.1107/S1600536810047069

**Published:** 2010-11-20

**Authors:** Christoffer Hamark, Jens Landström, Lars Eriksson, Göran Widmalm

**Affiliations:** a Department of Organic Chemistry, Arrhenius Laboratory, Stockholm University, S-106 91 Stockholm, Sweden; b Department of Environmental and Material Chemistry, Arrhenius Laboratory, Stockholm University, S-106 91 Stockholm, Sweden

## Abstract

In the title compound, C_30_H_31_NO_6_S, the plane of the *N*-phthalimido group is nearly orthogonal to the least-squares plane of the sugar ring (defined by atoms C2, C3, C5 and O5 using standard glucose nomenclature), making a dihedral angle of 72.8 (1)°. The thio­ethyl group has the *exo*-anomeric conformation. The hy­droxy group forms an inter­molecular hydrogen bond to the O atom in the sugar ring, generating [100] chains. There are four close π–π contacts with centroid–centroid distances less than 4.0 Å, all with dihedral angles between the inter­acting π systems of only ≃ 8°, supporting energetically favourable stacking inter­actions.

## Related literature

The title thio­glycoside is a valuable inter­mediate in synthesis of oligosaccharides containing *N*-acetyl-d-glucosa­mine residues, see: Söderman *et al.* (2002 ▶). For the *exo*-anomeric effect, see: Thøgersen *et al.* (1982 ▶). For total puckering amplitudes for previously described pyran­osides, see: Färnbäck *et al.* (2007 ▶). For the synthesis, see: Macindoe *et al.* (1995 ▶). 
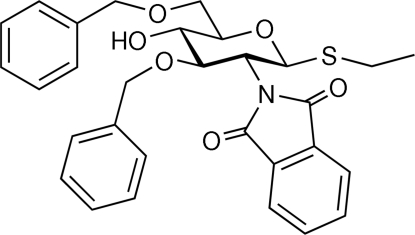


## Experimental

### 

#### Crystal data

C_30_H_31_NO_6_S
*M* *_r_* = 533.62
Orthorhombic, 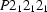

*a* = 8.5313 (1) Å
*b* = 14.7728 (2) Å
*c* = 21.1940 (4) Å
*V* = 2671.11 (7) Å^3^
*Z* = 4
Mo *K*α radiation
μ = 0.17 mm^−1^
*T* = 100 K
0.25 × 0.10 × 0.05 mm

#### Data collection

Oxford Diffraction Xcalibur II with Sapphire-3 CCD diffractometer
Absorption correction: multi-scan (*CrysAlis RED*; Oxford Diffraction, 2006 ▶) *T*_min_ = 0.96, *T*_max_ = 0.98
17362 measured reflections
5059 independent reflections
4023 reflections with *I* > 2σ(*I*)
*R*_int_ = 0.054

#### Refinement

*R*[*F* ^2^ > 2σ(*F* ^2^)] = 0.044
*wR*(*F* ^2^) = 0.067
*S* = 0.95
5059 reflections
346 parameters
H-atom parameters constrained
Δρ_max_ = 0.49 e Å^−3^
Δρ_min_ = −0.27 e Å^−3^
Absolute structure: Flack (1983) ▶, 2173 Friedel pairs
Flack parameter: 0.01 (7)

### 

Data collection: *CrysAlis CCD* (Oxford Diffraction, 2006 ▶); cell refinement: *CrysAlis RED* (Oxford Diffraction, 2006 ▶); data reduction: *CrysAlis RED*; program(s) used to solve structure: *SHELXS97* (Sheldrick, 2008 ▶); program(s) used to refine structure: *SHELXL97* (Sheldrick, 2008 ▶); molecular graphics: *DIAMOND* (Brandenburg, 1999 ▶); software used to prepare material for publication: *PLATON* (Spek, 2009 ▶).

## Supplementary Material

Crystal structure: contains datablocks global, I. DOI: 10.1107/S1600536810047069/nk2066sup1.cif

Structure factors: contains datablocks I. DOI: 10.1107/S1600536810047069/nk2066Isup2.hkl

Additional supplementary materials:  crystallographic information; 3D view; checkCIF report

## Figures and Tables

**Table:** 

S1—C1	1.796 (2)
C7—S1	1.819 (2)

**Table:** 

C1—S1—C7	99.10 (11)

**Table:** 

C7—S1—C1—H1	47.4
C7—S1—C1—O5	−72.06 (17)
O5—C5—C6—O6	63.3 (2)

**Table 2:** Hydrogen-bond geometry (Å, °)

*D*—H⋯*A*	*D*—H	H⋯*A*	*D*⋯*A*	*D*—H⋯*A*
O4—H4*A*⋯O5^i^	0.84	1.99	2.817 (2)	168

Symmetry code: (i) 
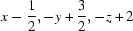
.

**Fig. 1.**
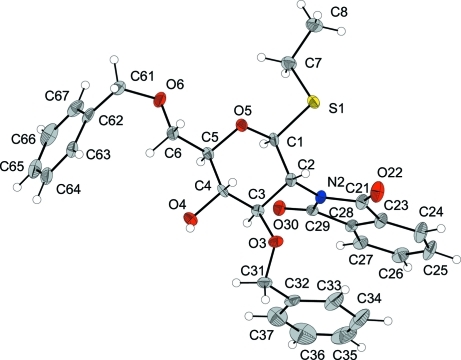
Molecular structure showing 50% probability displacement ellipsoids.

## supplementary crystallographic information

### Comment

The title thioglycoside is a valuable intermediate in synthesis of oligosaccharides containing *N*-acetyl-D-glucosamine residues (Söderman *et al.* 2002). In the structure the least square plane of the N-phthaloyl group makes a dihedral angle of 72.8 (1)° to the sugar ring plane defined by the four atoms (C2,C3,C5,O5). The conformation of the glycosidic torsion angle φ (H1—C1—S1—C7) is govered by the *exo*-anomeric effect (Thøgersen *et al.*, 1982) and also for this thioglucoside the torsion angles of 47.4° is typical of what is observed for glucosides with an oxygen atom at the glycosidic linkage. The Cremer-Pople parameters for the sugar ring (O5 → C5) are: Q=0.582 (2) Å, θ=12.5 (2)° and φ=310 (1)°. The Q-value is similar to total puckering amplitudes for previously described pyranosides (Färnbäck *et al.*, 2007).

The hydroxy group present in the title compound forms an intermolecular hydrogen bond with O5 in a neighbouring molecule, making up chains along the [100] direction. Besides this conventional hydrogen bond the intermolecular packing is stabilized by interactions between substituents of the sugar rings. There are four close π–π contacts with d(*Cg*—*Cg*) < 4.0 Å, all with dihedral angles between the interacting π systems of only ≈ 8° supporting energetically favourable stacking interactions.

### Experimental

The title compound (Macindoe *et al.*, 1995) was obtained from ethyl 4,6-*O*-benzylidene-2-deoxy-*N*-phtalimido-1-thio-β-D– glucopyranoside by bensylation of O3 and subsequent reductive opening of the 4,6-*O*-benzylidene group using NaCNBH_3_ and HCl(*g*) in tetrahydrofuran to give the 3,6-di-*O*-benzyl derivative. The title compound was crystallized from diethyl ether/pentane at ambient temperature to give colorless crystals.

### Refinement

The hydrogen atoms were positioned in calculated positions and refined in riding mode with C–H = 0.95–1.00 Å, O–H = 0.84 Å and *U*_iso_(H) = 1.2*U*_eq_(C) or *U*_iso_(H) = 1.5*U*_eq_(C,*O*) for methyl and hydroxy H atoms. The hydroxy H atom initial position was determined with a tetrahedral C—O—H angle and location such that it forms a favourable hydrogen bond to another oxygen atom. Finally the hydroxy H atoms were allowed to rotate about the C—O bond. The Flack parameter was determined to be 0.01 (7) from 2173 Friedel pairs.

### Crystal data

**Table:**

C_30_H_31_NO_6_S	*F*(000) = 1128
*M**_r_* = 533.62	*D*_x_ = 1.327 Mg m^−^^3^
Orthorhombic, *P*2_1_2_1_2_1_	Mo *K*α radiation, λ = 0.71073 Å
Hall symbol: P 2ac 2ab	Cell parameters from 6086 reflections
*a* = 8.5313 (1) Å	θ = 3.7–32.3°
*b* = 14.7728 (2) Å	µ = 0.17 mm^−^^1^
*c* = 21.1940 (4) Å	*T* = 100 K
*V* = 2671.11 (7) Å^3^	Prism, colourless
*Z* = 4	0.25 × 0.10 × 0.05 mm

### Data collection

**Table:**

Oxford Diffraction Xcalibur II with Sapphire-3 CCD diffractometer	5059 independent reflections
Radiation source: Enhance (Mo) X-ray Source	4023 reflections with *I* > 2σ(*I*)
graphite	*R*_int_ = 0.054
Detector resolution: 16.5467 pixels mm^-1^	θ_max_ = 25.7°, θ_min_ = 3.7°
ω scans at differnt φ	*h* = −10→10
Absorption correction: multi-scan (*CrysAlis RED*; Oxford Diffraction, 2006)	*k* = −13→18
*T*_min_ = 0.96, *T*_max_ = 0.98	*l* = −25→25
17362 measured reflections	

### Refinement

**Table:**

Refinement on *F*^2^	Hydrogen site location: inferred from neighbouring sites
Least-squares matrix: full	H-atom parameters constrained
*R*[*F*^2^ > 2σ(*F*^2^)] = 0.044	*w* = 1/[σ^2^(*F*_o_^2^) + (0.0261*P*)^2^] where *P* = (*F*_o_^2^ + 2*F*_c_^2^)/3
*wR*(*F*^2^) = 0.067	(Δ/σ)_max_ = 0.001
*S* = 0.95	Δρ_max_ = 0.49 e Å^−^^3^
5059 reflections	Δρ_min_ = −0.27 e Å^−^^3^
346 parameters	Extinction correction: *SHELXL97* (Sheldrick, 2008), Fc^*^=kFc[1+0.001xFc^2^λ^3^/sin(2θ)]^-1/4^
0 restraints	Extinction coefficient: 0.0028 (4)
Primary atom site location: structure-invariant direct methods	Absolute structure: Flack (1983), 2173 Friedel pairs
Secondary atom site location: difference Fourier map	Flack parameter: 0.01 (7)

### Special details

**Table:**

Experimental. CrysAlis RED, Oxford Diffraction Ltd.,Version 1.171.29.2. Empirical absorption correction using spherical harmonics, implemented in SCALE3 ABSPACK scaling algorithm.
Geometry. All e.s.d.'s (except the e.s.d. in the dihedral angle between two l.s. planes) are estimated using the full covariance matrix. The cell e.s.d.'s are taken into account individually in the estimation of e.s.d.'s in distances, angles and torsion angles; correlations between e.s.d.'s in cell parameters are only used when they are defined by crystal symmetry. An approximate (isotropic) treatment of cell e.s.d.'s is used for estimating e.s.d.'s involving l.s. planes.
Refinement. Refinement of *F*^2^ against ALL reflections. The weighted *R*-factor *wR* and goodness of fit *S* are based on *F*^2^, conventional *R*-factors *R* are based on *F*, with *F* set to zero for negative *F*^2^. The threshold expression of *F*^2^ > σ(*F*^2^) is used only for calculating *R*-factors(gt) *etc*. and is not relevant to the choice of reflections for refinement. *R*-factors based on *F*^2^ are statistically about twice as large as those based on *F*, and *R*- factors based on ALL data will be even larger.

### Fractional atomic coordinates and isotropic or equivalent isotropic displacement parameters (Å^2^)

**Table:**

	*x*	*y*	*z*	*U*_iso_*/*U*_eq_	
S1	0.32681 (7)	0.66068 (4)	0.88067 (3)	0.01942 (15)	
C7	0.3666 (3)	0.73807 (17)	0.81586 (11)	0.0255 (6)	
H7A	0.3252	0.7127	0.7760	0.031*	
H7B	0.3142	0.7968	0.8237	0.031*	
C8	0.5419 (3)	0.75202 (18)	0.81042 (12)	0.0301 (6)	
H8A	0.5822	0.7771	0.8500	0.045*	
H8B	0.5639	0.7941	0.7758	0.045*	
H8C	0.5929	0.6939	0.8019	0.045*	
C1	0.1170 (2)	0.66968 (15)	0.88275 (11)	0.0156 (5)	
H1	0.0741	0.6638	0.8390	0.019*	
C2	0.0500 (3)	0.59407 (14)	0.92453 (10)	0.0149 (5)	
H2	0.1098	0.5946	0.9651	0.018*	
C3	−0.1227 (3)	0.60855 (14)	0.94058 (11)	0.0147 (5)	
H3	−0.1889	0.5930	0.9032	0.018*	
C4	−0.1537 (3)	0.70604 (14)	0.96039 (11)	0.0150 (5)	
H4	−0.1002	0.7184	1.0015	0.018*	
C5	−0.0901 (3)	0.76978 (15)	0.91011 (11)	0.0153 (5)	
H5	−0.1369	0.7540	0.8683	0.018*	
O5	0.07730 (18)	0.75651 (10)	0.90779 (7)	0.0171 (4)	
C6	−0.1223 (3)	0.86722 (14)	0.92464 (11)	0.0195 (6)	
H6A	−0.2366	0.8767	0.9293	0.023*	
H6B	−0.0711	0.8841	0.9649	0.023*	
N2	0.0742 (2)	0.50558 (12)	0.89529 (8)	0.0144 (4)	
C21	0.1697 (3)	0.43819 (15)	0.92183 (11)	0.0206 (6)	
O22	0.2428 (2)	0.44655 (11)	0.97041 (8)	0.0297 (4)	
C23	0.1603 (3)	0.36067 (14)	0.87817 (12)	0.0203 (5)	
C24	0.2295 (3)	0.27621 (16)	0.88227 (12)	0.0295 (6)	
H24	0.2966	0.2608	0.9164	0.035*	
C25	0.1966 (3)	0.21488 (16)	0.83440 (12)	0.0315 (7)	
H25	0.2401	0.1558	0.8364	0.038*	
C26	0.1023 (3)	0.23773 (16)	0.78409 (12)	0.0265 (6)	
H26	0.0831	0.1944	0.7519	0.032*	
C27	0.0348 (3)	0.32341 (15)	0.77964 (11)	0.0211 (6)	
H27	−0.0290	0.3399	0.7447	0.025*	
C28	0.0649 (3)	0.38319 (15)	0.82828 (11)	0.0165 (5)	
C29	0.0087 (3)	0.47748 (15)	0.83788 (11)	0.0152 (5)	
O30	−0.07326 (19)	0.52424 (10)	0.80436 (7)	0.0210 (4)	
O3	−0.15948 (18)	0.54906 (10)	0.99189 (7)	0.0198 (4)	
C31	−0.3063 (3)	0.50213 (15)	0.98636 (11)	0.0223 (6)	
H31A	−0.3821	0.5411	0.9637	0.027*	
H31B	−0.3487	0.4903	1.0291	0.027*	
C32	−0.2895 (3)	0.41349 (15)	0.95149 (11)	0.0223 (6)	
C33	−0.1825 (3)	0.34987 (17)	0.97248 (13)	0.0380 (7)	
H33	−0.1169	0.3628	1.0076	0.046*	
C34	−0.1718 (4)	0.26677 (19)	0.94180 (16)	0.0544 (10)	
H34	−0.1010	0.2221	0.9569	0.065*	
C35	−0.2628 (4)	0.2490 (2)	0.88986 (18)	0.0575 (10)	
H35	−0.2541	0.1925	0.8687	0.069*	
C36	−0.3653 (4)	0.3123 (2)	0.86889 (16)	0.0570 (10)	
H36	−0.4277	0.3003	0.8327	0.068*	
C37	−0.3799 (3)	0.3945 (2)	0.89988 (13)	0.0406 (8)	
H37	−0.4532	0.4381	0.8851	0.049*	
O4	−0.31735 (17)	0.71949 (11)	0.96734 (7)	0.0214 (4)	
H4A	−0.3403	0.7205	1.0059	0.032*	
O6	−0.06402 (19)	0.92258 (10)	0.87509 (8)	0.0278 (4)	
C61	−0.1367 (3)	1.01026 (15)	0.87450 (13)	0.0273 (6)	
H61A	−0.0701	1.0527	0.8503	0.033*	
H61B	−0.1443	1.0332	0.9183	0.033*	
C62	−0.2970 (3)	1.00801 (15)	0.84587 (11)	0.0215 (6)	
C63	−0.4318 (3)	0.99598 (14)	0.88219 (12)	0.0211 (5)	
H63	−0.4234	0.9938	0.9269	0.025*	
C64	−0.5772 (3)	0.98725 (15)	0.85426 (12)	0.0274 (6)	
H64	−0.6670	0.9767	0.8797	0.033*	
C65	−0.5930 (3)	0.99374 (16)	0.78969 (12)	0.0301 (7)	
H65	−0.6932	0.9880	0.7705	0.036*	
C66	−0.4620 (4)	1.00867 (17)	0.75332 (13)	0.0368 (7)	
H66	−0.4723	1.0143	0.7089	0.044*	
C67	−0.3158 (3)	1.01558 (15)	0.78078 (12)	0.0305 (7)	
H67	−0.2266	1.0257	0.7549	0.037*	

### Atomic displacement parameters (Å^2^)

**Table:**

	*U*^11^	*U*^22^	*U*^33^	*U*^12^	*U*^13^	*U*^23^
S1	0.0140 (3)	0.0220 (3)	0.0222 (3)	0.0024 (3)	0.0021 (3)	0.0003 (3)
C7	0.0210 (16)	0.0300 (15)	0.0256 (15)	−0.0020 (12)	0.0010 (11)	0.0062 (12)
C8	0.0232 (16)	0.0318 (15)	0.0353 (17)	−0.0039 (13)	0.0063 (12)	0.0013 (13)
C1	0.0131 (12)	0.0157 (12)	0.0180 (13)	0.0045 (10)	−0.0022 (10)	−0.0044 (11)
C2	0.0171 (13)	0.0156 (13)	0.0118 (13)	0.0027 (11)	−0.0043 (10)	−0.0024 (10)
C3	0.0166 (14)	0.0183 (13)	0.0091 (13)	−0.0013 (10)	−0.0003 (10)	−0.0001 (10)
C4	0.0081 (13)	0.0219 (13)	0.0148 (13)	0.0027 (11)	−0.0007 (10)	−0.0028 (10)
C5	0.0132 (14)	0.0177 (13)	0.0151 (13)	0.0018 (10)	−0.0007 (10)	−0.0032 (10)
O5	0.0155 (9)	0.0154 (9)	0.0203 (9)	0.0016 (7)	0.0001 (7)	−0.0039 (7)
C6	0.0176 (14)	0.0193 (14)	0.0217 (14)	0.0040 (11)	0.0015 (11)	0.0014 (11)
N2	0.0189 (11)	0.0129 (10)	0.0114 (10)	0.0031 (9)	−0.0011 (8)	−0.0028 (8)
C21	0.0265 (15)	0.0184 (13)	0.0168 (14)	0.0034 (13)	0.0000 (12)	0.0002 (11)
O22	0.0424 (12)	0.0276 (10)	0.0190 (10)	0.0120 (9)	−0.0102 (9)	−0.0042 (8)
C23	0.0293 (14)	0.0155 (13)	0.0162 (13)	0.0038 (11)	0.0019 (12)	−0.0029 (11)
C24	0.0447 (17)	0.0248 (14)	0.0190 (14)	0.0126 (13)	−0.0043 (14)	−0.0001 (13)
C25	0.0480 (19)	0.0146 (13)	0.0319 (16)	0.0104 (13)	0.0022 (14)	−0.0001 (12)
C26	0.0360 (17)	0.0201 (15)	0.0236 (15)	−0.0035 (13)	0.0041 (13)	−0.0070 (12)
C27	0.0261 (15)	0.0193 (14)	0.0180 (14)	−0.0025 (12)	0.0007 (11)	−0.0021 (11)
C28	0.0211 (14)	0.0149 (13)	0.0135 (13)	0.0005 (11)	0.0049 (11)	0.0012 (10)
C29	0.0139 (13)	0.0159 (13)	0.0159 (13)	−0.0005 (10)	0.0036 (11)	0.0008 (11)
O30	0.0234 (10)	0.0190 (9)	0.0205 (10)	0.0025 (8)	−0.0060 (8)	−0.0023 (7)
O3	0.0218 (9)	0.0205 (9)	0.0172 (9)	−0.0045 (8)	0.0014 (7)	0.0037 (7)
C31	0.0197 (14)	0.0204 (13)	0.0269 (14)	−0.0048 (12)	0.0062 (11)	0.0018 (11)
C32	0.0269 (16)	0.0179 (13)	0.0221 (14)	−0.0060 (12)	0.0077 (12)	0.0028 (11)
C33	0.056 (2)	0.0241 (16)	0.0342 (17)	−0.0014 (15)	0.0080 (15)	0.0051 (13)
C34	0.083 (3)	0.0226 (17)	0.057 (2)	0.0080 (18)	0.032 (2)	0.0126 (16)
C35	0.075 (3)	0.0244 (17)	0.073 (3)	−0.0148 (17)	0.031 (2)	−0.0201 (19)
C36	0.057 (2)	0.064 (2)	0.051 (2)	−0.0161 (19)	−0.0022 (18)	−0.0312 (19)
C37	0.0401 (19)	0.0418 (18)	0.0397 (19)	−0.0019 (14)	−0.0013 (15)	−0.0122 (14)
O4	0.0150 (9)	0.0266 (9)	0.0225 (9)	0.0008 (8)	0.0042 (8)	−0.0009 (8)
O6	0.0289 (10)	0.0167 (9)	0.0377 (11)	0.0066 (8)	0.0098 (9)	0.0046 (8)
C61	0.0272 (16)	0.0168 (14)	0.0378 (17)	0.0014 (11)	0.0061 (13)	0.0024 (12)
C62	0.0341 (16)	0.0085 (12)	0.0221 (14)	0.0057 (12)	0.0075 (12)	−0.0010 (10)
C63	0.0266 (15)	0.0178 (13)	0.0189 (13)	0.0078 (12)	0.0016 (13)	0.0005 (12)
C64	0.0289 (16)	0.0210 (15)	0.0325 (16)	0.0097 (13)	0.0042 (13)	0.0024 (12)
C65	0.0388 (18)	0.0205 (14)	0.0311 (16)	0.0077 (14)	−0.0114 (14)	−0.0002 (13)
C66	0.066 (2)	0.0267 (16)	0.0182 (15)	0.0091 (16)	−0.0061 (15)	−0.0012 (12)
C67	0.0456 (19)	0.0170 (14)	0.0289 (16)	0.0037 (14)	0.0160 (14)	0.0012 (12)

### Geometric parameters (Å, °)

**Table:**

S1—C1	1.796 (2)	C26—H26	0.9500
S1—C7	1.819 (2)	C27—C28	1.381 (3)
C7—C8	1.513 (3)	C27—H27	0.9500
C7—H7A	0.9900	C28—C29	1.487 (3)
C7—H7B	0.9900	C29—O30	1.213 (3)
C8—H8A	0.9800	O3—C31	1.436 (3)
C8—H8B	0.9800	C31—C32	1.510 (3)
C8—H8C	0.9800	C31—H31A	0.9900
C1—O5	1.429 (2)	C31—H31B	0.9900
C1—C2	1.536 (3)	C32—C37	1.367 (4)
C1—H1	1.0000	C32—C33	1.383 (3)
C2—N2	1.462 (3)	C33—C34	1.392 (4)
C2—C3	1.526 (3)	C33—H33	0.9500
C2—H2	1.0000	C34—C35	1.373 (5)
C3—O3	1.433 (3)	C34—H34	0.9500
C3—C4	1.523 (3)	C35—C36	1.356 (4)
C3—H3	1.0000	C35—H35	0.9500
C4—O4	1.417 (2)	C36—C37	1.386 (4)
C4—C5	1.522 (3)	C36—H36	0.9500
C4—H4	1.0000	C37—H37	0.9500
C5—O5	1.442 (3)	O4—H4A	0.8400
C5—C6	1.498 (3)	O6—C61	1.436 (3)
C5—H5	1.0000	C61—C62	1.496 (3)
C6—O6	1.421 (3)	C61—H61A	0.9900
C6—H6A	0.9900	C61—H61B	0.9900
C6—H6B	0.9900	C62—C67	1.393 (3)
N2—C29	1.402 (3)	C62—C63	1.395 (3)
N2—C21	1.404 (3)	C63—C64	1.381 (3)
C21—O22	1.210 (3)	C63—H63	0.9500
C21—C23	1.474 (3)	C64—C65	1.378 (3)
C23—C28	1.376 (3)	C64—H64	0.9500
C23—C24	1.383 (3)	C65—C66	1.376 (4)
C24—C25	1.389 (3)	C65—H65	0.9500
C24—H24	0.9500	C66—C67	1.380 (4)
C25—C26	1.377 (3)	C66—H66	0.9500
C25—H25	0.9500	C67—H67	0.9500
C26—C27	1.394 (3)		
			
C1—S1—C7	99.10 (11)	C24—C25—H25	119.2
C8—C7—S1	109.13 (17)	C25—C26—C27	121.1 (2)
C8—C7—H7A	109.9	C25—C26—H26	119.5
S1—C7—H7A	109.9	C27—C26—H26	119.5
C8—C7—H7B	109.9	C28—C27—C26	117.0 (2)
S1—C7—H7B	109.9	C28—C27—H27	121.5
H7A—C7—H7B	108.3	C26—C27—H27	121.5
C7—C8—H8A	109.5	C23—C28—C27	121.9 (2)
C7—C8—H8B	109.5	C23—C28—C29	108.2 (2)
H8A—C8—H8B	109.5	C27—C28—C29	129.9 (2)
C7—C8—H8C	109.5	O30—C29—N2	124.7 (2)
H8A—C8—H8C	109.5	O30—C29—C28	129.7 (2)
H8B—C8—H8C	109.5	N2—C29—C28	105.5 (2)
O5—C1—C2	110.52 (17)	C3—O3—C31	115.16 (17)
O5—C1—S1	108.16 (14)	O3—C31—C32	112.04 (18)
C2—C1—S1	109.35 (15)	O3—C31—H31A	109.2
O5—C1—H1	109.6	C32—C31—H31A	109.2
C2—C1—H1	109.6	O3—C31—H31B	109.2
S1—C1—H1	109.6	C32—C31—H31B	109.2
N2—C2—C3	110.89 (18)	H31A—C31—H31B	107.9
N2—C2—C1	110.68 (18)	C37—C32—C33	119.4 (2)
C3—C2—C1	112.68 (17)	C37—C32—C31	121.0 (2)
N2—C2—H2	107.4	C33—C32—C31	119.6 (2)
C3—C2—H2	107.4	C32—C33—C34	119.5 (3)
C1—C2—H2	107.4	C32—C33—H33	120.3
O3—C3—C4	109.41 (17)	C34—C33—H33	120.3
O3—C3—C2	107.13 (17)	C35—C34—C33	120.4 (3)
C4—C3—C2	111.22 (18)	C35—C34—H34	119.8
O3—C3—H3	109.7	C33—C34—H34	119.8
C4—C3—H3	109.7	C36—C35—C34	119.7 (3)
C2—C3—H3	109.7	C36—C35—H35	120.1
O4—C4—C5	109.72 (18)	C34—C35—H35	120.1
O4—C4—C3	109.43 (18)	C35—C36—C37	120.5 (3)
C5—C4—C3	109.25 (17)	C35—C36—H36	119.8
O4—C4—H4	109.5	C37—C36—H36	119.8
C5—C4—H4	109.5	C32—C37—C36	120.5 (3)
C3—C4—H4	109.5	C32—C37—H37	119.7
O5—C5—C6	108.63 (19)	C36—C37—H37	119.7
O5—C5—C4	107.04 (18)	C4—O4—H4A	109.5
C6—C5—C4	112.65 (18)	C6—O6—C61	111.98 (18)
O5—C5—H5	109.5	O6—C61—C62	112.21 (19)
C6—C5—H5	109.5	O6—C61—H61A	109.2
C4—C5—H5	109.5	C62—C61—H61A	109.2
C1—O5—C5	111.66 (16)	O6—C61—H61B	109.2
O6—C6—C5	109.69 (18)	C62—C61—H61B	109.2
O6—C6—H6A	109.7	H61A—C61—H61B	107.9
C5—C6—H6A	109.7	C67—C62—C63	117.5 (2)
O6—C6—H6B	109.7	C67—C62—C61	120.4 (2)
C5—C6—H6B	109.7	C63—C62—C61	122.2 (2)
H6A—C6—H6B	108.2	C64—C63—C62	121.1 (2)
C29—N2—C21	111.67 (18)	C64—C63—H63	119.5
C29—N2—C2	125.19 (18)	C62—C63—H63	119.5
C21—N2—C2	123.14 (18)	C65—C64—C63	120.5 (3)
O22—C21—N2	124.6 (2)	C65—C64—H64	119.8
O22—C21—C23	129.9 (2)	C63—C64—H64	119.8
N2—C21—C23	105.5 (2)	C66—C65—C64	119.2 (3)
C28—C23—C24	121.3 (2)	C66—C65—H65	120.4
C28—C23—C21	109.05 (19)	C64—C65—H65	120.4
C24—C23—C21	129.7 (2)	C65—C66—C67	120.7 (2)
C23—C24—C25	117.2 (2)	C65—C66—H66	119.7
C23—C24—H24	121.4	C67—C66—H66	119.7
C25—C24—H24	121.4	C66—C67—C62	121.1 (2)
C26—C25—C24	121.6 (2)	C66—C67—H67	119.5
C26—C25—H25	119.2	C62—C67—H67	119.5
			
C7—S1—C1—H1	47.4	C24—C25—C26—C27	−0.6 (4)
C1—S1—C7—C8	172.39 (18)	C25—C26—C27—C28	−1.0 (4)
C7—S1—C1—O5	−72.06 (17)	C24—C23—C28—C27	−0.8 (4)
C7—S1—C1—C2	167.52 (16)	C21—C23—C28—C27	179.8 (2)
O5—C1—C2—N2	173.35 (17)	C24—C23—C28—C29	179.6 (2)
S1—C1—C2—N2	−67.7 (2)	C21—C23—C28—C29	0.2 (3)
O5—C1—C2—C3	48.5 (2)	C26—C27—C28—C23	1.7 (4)
S1—C1—C2—C3	167.49 (15)	C26—C27—C28—C29	−178.8 (2)
N2—C2—C3—O3	70.3 (2)	C21—N2—C29—O30	−177.1 (2)
C1—C2—C3—O3	−164.98 (17)	C2—N2—C29—O30	2.2 (3)
N2—C2—C3—C4	−170.16 (17)	C21—N2—C29—C28	1.4 (2)
C1—C2—C3—C4	−45.5 (2)	C2—N2—C29—C28	−179.31 (19)
O3—C3—C4—O4	−69.0 (2)	C23—C28—C29—O30	177.4 (2)
C2—C3—C4—O4	172.88 (17)	C27—C28—C29—O30	−2.1 (4)
O3—C3—C4—C5	170.90 (17)	C23—C28—C29—N2	−1.0 (2)
C2—C3—C4—C5	52.7 (2)	C27—C28—C29—N2	179.5 (2)
O4—C4—C5—O5	176.83 (17)	C4—C3—O3—C31	102.6 (2)
C3—C4—C5—O5	−63.2 (2)	C2—C3—O3—C31	−136.77 (18)
O4—C4—C5—C6	57.5 (2)	C3—O3—C31—C32	88.6 (2)
C3—C4—C5—C6	177.45 (19)	O3—C31—C32—C37	−125.6 (3)
C2—C1—O5—C5	−61.2 (2)	O3—C31—C32—C33	55.3 (3)
S1—C1—O5—C5	179.08 (15)	C37—C32—C33—C34	−1.8 (4)
C6—C5—O5—C1	−169.14 (17)	C31—C32—C33—C34	177.4 (2)
C4—C5—O5—C1	69.0 (2)	C32—C33—C34—C35	2.1 (4)
O5—C5—C6—O6	63.3 (2)	C33—C34—C35—C36	−0.9 (5)
C4—C5—C6—O6	−178.31 (19)	C34—C35—C36—C37	−0.6 (5)
C3—C2—N2—C29	62.2 (3)	C33—C32—C37—C36	0.3 (4)
C1—C2—N2—C29	−63.6 (3)	C31—C32—C37—C36	−178.9 (3)
C3—C2—N2—C21	−118.6 (2)	C35—C36—C37—C32	0.9 (5)
C1—C2—N2—C21	115.6 (2)	C5—C6—O6—C61	159.61 (19)
C29—N2—C21—O22	178.0 (2)	C6—O6—C61—C62	−78.3 (3)
C2—N2—C21—O22	−1.3 (4)	O6—C61—C62—C67	−85.7 (3)
C29—N2—C21—C23	−1.3 (3)	O6—C61—C62—C63	92.6 (3)
C2—N2—C21—C23	179.4 (2)	C67—C62—C63—C64	3.3 (3)
O22—C21—C23—C28	−178.6 (3)	C61—C62—C63—C64	−175.0 (2)
N2—C21—C23—C28	0.7 (3)	C62—C63—C64—C65	−2.5 (3)
O22—C21—C23—C24	2.1 (5)	C63—C64—C65—C66	0.3 (4)
N2—C21—C23—C24	−178.7 (2)	C64—C65—C66—C67	1.1 (4)
C28—C23—C24—C25	−0.9 (4)	C65—C66—C67—C62	−0.3 (4)
C21—C23—C24—C25	178.4 (3)	C63—C62—C67—C66	−1.9 (3)
C23—C24—C25—C26	1.6 (4)	C61—C62—C67—C66	176.4 (2)

### Hydrogen-bond geometry (Å, °)

**Table:**

*D*—H···*A*	*D*—H	H···*A*	*D*···*A*	*D*—H···*A*
O4—H4A···O5^i^	0.84	1.99	2.817 (2)	168

Symmetry codes: (i) *x*−1/2, −*y*+3/2, −*z*+2.
